# Notes and News

**Published:** 1905-12-09

**Authors:** 


### Notes and News.

Princess Henry of Battenberg opened on Friday the new Hammersmith Workhouse and Infirmary which have been erected in Ducane Road, Wormwood Scrubs. The Infirmary blocks are divided into wards, each of which contains 24 beds, in addition to which there are the necessary administrative departments and a separate nurses' home. The latter has already been described in The Hospital. The cost of the building, without the site, is £207,000.

The eminent Japanese surgeon, Baron Tagani, formerly Surgeon-General to the Japanese Army, will commence on December 26 a series of lectures in America on military sanitation. He is afterwards to visit England for the purpose of arranging for the affiliation of the Japanese Medical Colleges with the British Medical College, and he is sure of a warm welcome.

The death of Staff Surgeon-General Professor Dr. Von Leuthold, physician-in-ordinary to the German Emperor, from influenza is announced. He held that post during the last three years of the reign of the Emperor William I., and was confirmed in his appointment by the present Emperor. At the time of his death he was Surgeon-General-in-Chief of the Prussian Army, and he rendered valuable service in the domain of military hygiene.

The powers granted to the recently-appointed Poor-Law Commission were officially announced on Tuesday night. The Commission can call such persons as they judge likely to afford any information upon the subject, and call for information in writing, and for all such books, documents, registers, and records as they think necessary. They are also empowered to personally inspect such places as they deem expedient. If the Commissioners think proper they can report their proceedings from time to time.

The late Mr. John Edward Taylor, proprietor of the *Manchester Guardian*, has left the sum of £5,000 to the London Temperance Hospital, and £1,000 to the Manchester Infirmary.

The annual Conference of the Pharmaceutical Society of Great Britain will be held in Birmingham in the week beginning July 23. Two decades will then have elapsed since the Conference last met in Birmingham.

A children's ward has just been opened at the Royal Boscombe and West Hants Hospital, Bournemouth, erected at a cost of about £2,000. An anonymous donation of £1,076, in memory of a son, was announced during the proceedings.

An exhibition and sale of water-colour sketches of English cathedrals, by Mrs. Mostyn, in aid of the League of Mercy, was opened on Tuesday at the Ryder Gallery, 47 Albemarle Street. Mrs. Mostyn is Vice-President of the Chelsea branch, and this is the second year she has organised it for the same object.

Several hospitals receive bequests under the will of the late Mrs. Eliza Eyre, of Kingsmill, Dursley. The testator left £2,000 each to Guy's Hospital, St. George's Hospital, Gloucester General Infirmary, and the Royal Sea-Bathing Hospital at Margate; £1,000 each to the London Hospital, Bristol Royal Infirmary, and Bristol General Hospital; and £500 each to Gloucester Free Hospital for Women, the West of England Sanatorium at Weston-super-Mare, and the Convalescent Hospital at Walton-by-Clevedon.

The charities in the Isle of Man will benefit very largely under the will of the late Mr. Henry Bloom Noble, of Douglas. Acting in accordance with the provisions of his will, the trustees have allocated £20,000 for the purpose of building a new hospital in Douglas, £1,500 for repairing the present Douglas Hospital, and £6,000 for a cottage hospital at Ramsey. The Liverpool Royal Infirmary is also to receive £2,500 for the maintenance of a bed; and the trustees have also arranged for beds in Kelling Sanatorium for Consumptives.

The deputation from the Royal Commission on the care and control of the Feeble-minded, who went to the United States to investigate the methods of dealing with the mentally deficient in that country, arrived in Liverpool on Sunday. Mr. W. P. Byrne, C.B., one of the deputation, states that many features of American institutions for dealing with the feeble-minded are unknown in England, but the general opinion of the deputation is that, while much might be learned from the American system in the treatment of adults, the English methods of dealing with children are much superior to the American.

